# Assessment of a New Web-Based Sexual Concurrency Measurement Tool for Men Who Have Sex With Men

**DOI:** 10.2196/jmir.3211

**Published:** 2014-11-10

**Authors:** Eli S Rosenberg, Richard B Rothenberg, David G Kleinbaum, Rob B Stephenson, Patrick S Sullivan

**Affiliations:** ^1^Department of EpidemiologyEmory University Rollins School of Public HealthAtlanta, GAUnited States; ^2^Institute of Public HealthGeorgia State UniversityAtlanta, GAUnited States; ^3^Hubert Department of Global HealthEmory University Rollins School of Public HealthAtlanta, GAUnited States

**Keywords:** HIV, sexual networks, questionnaires, concurrency, MSM, sexual network measurement, online questionnaire

## Abstract

**Background:**

Men who have sex with men (MSM) are the most affected risk group in the United States’ human immunodeficiency virus (HIV) epidemic. Sexual concurrency, the overlapping of partnerships in time, accelerates HIV transmission in populations and has been documented at high levels among MSM. However, concurrency is challenging to measure empirically and variations in assessment techniques used (primarily the date overlap and direct question approaches) and the outcomes derived from them have led to heterogeneity and questionable validity of estimates among MSM and other populations.

**Objective:**

The aim was to evaluate a novel Web-based and interactive partnership-timing module designed for measuring concurrency among MSM, and to compare outcomes measured by the partnership-timing module to those of typical approaches in an online study of MSM.

**Methods:**

In an online study of MSM aged ≥18 years, we assessed concurrency by using the direct question method and by gathering the dates of first and last sex, with enhanced programming logic, for each reported partner in the previous 6 months. From these methods, we computed multiple concurrency cumulative prevalence outcomes: direct question, day resolution / date overlap, and month resolution / date overlap including both 1-month ties and excluding ties. We additionally computed variants of the UNAIDS point prevalence outcome. The partnership-timing module was also administered. It uses an interactive month resolution calendar to improve recall and follow-up questions to resolve temporal ambiguities, combines elements of the direct question and date overlap approaches. The agreement between the partnership-timing module and other concurrency outcomes was assessed with percent agreement, kappa statistic (κ), and matched odds ratios at the individual, dyad, and triad levels of analysis.

**Results:**

Among 2737 MSM who completed the partnership section of the partnership-timing module, 41.07% (1124/2737) of individuals had concurrent partners in the previous 6 months. The partnership-timing module had the highest degree of agreement with the direct question. Agreement was lower with date overlap outcomes (agreement range 79%-81%, κ range .55-.59) and lowest with the UNAIDS outcome at 5 months before interview (65% agreement, κ=.14, 95% CI .12-.16). All agreements declined after excluding individuals with 1 sex partner (always classified as not engaging in concurrency), although the highest agreement was still observed with the direct question technique (81% agreement, κ=.59, 95% CI .55-.63). Similar patterns in agreement were observed with dyad- and triad-level outcomes.

**Conclusions:**

The partnership-timing module showed strong concurrency detection ability and agreement with previous measures. These levels of agreement were greater than others have reported among previous measures. The partnership-timing module may be well suited to quantifying concurrency among MSM at multiple levels of analysis.

## Introduction

### Background

Men who have sex with men (MSM) have long been the most heavily impacted risk group in the United States’ human immunodeficiency virus (HIV) epidemic [1]. In 2010, MSM accounted for an estimated 66% of new HIV infections in the United States; since 2000, MSM have been the only transmission group for whom incidence has been increasing [2,3]. Emerging evidence suggests that the biological realities of differential transmission probabilities for anal and vaginal sex and heterosexual role segregation play a larger role in the HIV incidence disparities between MSM and heterosexuals than do differences in individual-level risk behavior [4-6]. The role of differential network-level factors may also be important, yet this remains insufficiently explored [6,7].

### Concurrency Accelerates HIV Transmission but Measurement Varies

One such factor is sexual concurrency, defined as “overlapping sexual partnerships where sexual intercourse with 1 partner occurs between 2 acts of intercourse with another partner” [8]. Concurrency has the potential to catalyze transmission in populations by increasing both sexual network connectivity and the likelihood of transmission during acute HIV infection [9,10]. Simulation-based, couples-based, and ecological studies have provided theoretical and empirical evidence of concurrency’s causal role in amplifying HIV epidemics [11-14].

Differences in the level and patterns of sexual concurrency between MSM and heterosexuals in the United States remain insufficiently understood. High levels of concurrent sex have been recently documented among MSM in the United States (18%-78% prevalence in previous year) [7,15,16], substantially greater than among heterosexual men (10%-11% in previous year) [7,17]. These reports all used differing methods of measuring concurrency, a common issue in concurrency research [18,19]. To properly describe the role concurrency might play in transmission among MSM, an improved understanding of the appropriateness of concurrency measures is needed.

It is important to differentiate between the tools used to elucidate sexual timing information and the concurrency measures derived from these tools because these 2 notions are subject to different limitations that have been conflated in critical examinations of concurrency measurement [8,20,21]. Two approaches, date overlap and direct question, are primarily used to gather concurrency responses, both of which involve assessment on a partner-by-partner basis for a given number of recent sex partners. On the other hand, a variety of individual-level concurrency measures have been calculated using data from these approaches.

### Date Overlap Method

In the date overlap method, the dates of first and last sex with each partner are gathered with the purpose of inspecting for overlapping partner intervals. Although seemingly powerful and precise if exact dates are used, this approach is subject to poor date recall and missing or illogical responses [20,22,23]. Variants of this measurement technique intended to alleviate these issues have been to gather date information at the month/year level only and as the number of days/weeks/month/years preceding the interview [8,17]. These alternatives come with potential temporal ambiguities for single-month interval overlaps (“ties”), which may be more common in populations with more short-term partnerships.

From these date collection techniques, multiple individual-level concurrency cumulative prevalence measures have been employed: having any exact date overlaps [24], any month resolution overlaps and including ties as concurrent [20,21,23,25], and, most commonly, any date overlaps but conservatively excluding ties [8,17,21,25]. These have been typically computed for a 12-month recall period.

The Joint United Nations Programme on HIV/AIDS (UNAIDS) working group has introduced a measure of concurrency, the point prevalence of concurrency at 6 months before interview, to be calculated as a month resolution overlap during this month and excluding ties [8,19]. This measure was chosen to emphasize longer-term relationships and overlaps, which are expected to contribute more greatly to the risk of concurrency in the sub-Saharan African context for which the measure was developed [8,19]. Yet this also creates the potential to drastically undercount the occurrence of concurrency in a population with frequent short-term sexual contacts, resulting in low sensitivity for screening those who engage in concurrent sexual partnerships.

### Direct Question Method

The direct question data collection method assesses, for each partnership, how many other sex partners were had during that partnership in the recall period. An individual-level period prevalence measure is then derived from inspection for any partnership with 1 or more outside partner [23]. This method is simple to administer, may be easier for recall, typically yields fewer missing data, and is less limited by the total partners able to be described in the survey [20,26]. Yet it is potentially impacted more by biases related to social desirability and in the perception of concurrency [21].

The few published comparisons have shown varied performance of these measures, partly due to the differences and limitations discussed. Nelson et al [20] found similar levels of concurrency among US heterosexuals, but only fair agreement, using month resolution date overlap (inclusive of ties) and direct question measures. Glynn et al [21] found lower agreement across a broader set of these measures and the most concurrency per direct question in Malawian heterosexuals. Maughan-Brown and Venkataramani [26] have reported similar findings in a South African comparison of the direct question and UNAIDS measures. Because no gold-standard method exists, it is unclear if the highest levels of concurrency measured by the direct method correspond to best detection.

### Levels of Analysis Are Important but Seldom Considered

**Figure 1:**
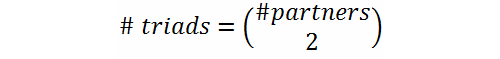
The number of possible triads among an individual’s sex partners.

Absent from previous discussions of concurrency measurement techniques are considerations of which levels of analysis they enable. Individual-level concurrency is important for the surveillance of those who engage in concurrent sex. Yet it offers a limited analytical perspective for the research purposes of empirically understanding the types, correlates, and implications of concurrency. This is because the fundamental unit at which concurrency operates is the triad, composed of an individual and 2 sex partners [27]. Individuals may contribute multiple triads (see Figure 1), and summarizing triads to form individual-level measures discards information about the partnership-level factors associated with concurrency. Recently published triadic results have described the prevalence of unprotected sex with both members among concurrent triads and the association between triadic concurrency and unprotected sex [15,28]. Of these measures, only those based on cumulative date overlap data permit triadic analysis.

The dyadic, or partner, perspective is another important level for understanding concurrency [29]. An individual’s concurrency does not impact one’s own risk of infection acquisition, but rather that of one’s partners, a distinction that has long stymied empirical analyses of concurrency [27,30]. Ideally, empirical analyses of infection risk due to concurrency would consider the types of partners involved and would quantify partners’ increased exposure and/or infection due to concurrent sex. We recently assessed such increased dyadic exposure among MSM [31]. Both date overlap and direct question approaches can be used to measure dyadic concurrency, although the latter is limited by the absence of data on other partners with whom the respondent was concurrent. UNAIDS-type point prevalence measures are insufficient for triadic and dyadic analyses because they are designed to detect only a subset of concurrent partnerships.

### Challenges in Measuring Concurrency Among MSM and the Need for Appropriate Tools

The majority of empirical concurrency measurement research has been in sub-Saharan African [21,25,32,33] and US heterosexual [23,34,35] settings, rather than among MSM, whose partnership patterns are distinct from these populations [7]. Compared to heterosexuals, MSM report more shorter-term casual partners on average [7,36]. This presents several challenges to concurrency measurement among MSM. First, to the extent that these partnerships are 1-time or are contained within a single month, substantial misclassification would be likely if month resolution date overlap measures are used, with disparate results seen depending on the inclusion of ties. Because MSM are more likely to report more than 1 sex partner and, thus, have more opportunity for concurrency, fewer individuals would be automatically classified as nonconcurrent by all measures compared to heterosexuals. This would be expected to result in higher estimated concurrency prevalence among MSM and a lower agreement between concurrency measures.

In this paper, we describe a novel, Web-based concurrency measurement tool used in 2 recent analyses [15,31]. It is designed to remedy reporting biases, enables triadic and dyadic analyses, and is tailored to the sexual activity patterns of MSM. This tool employs a compromise between date overlap and direct question methods, and is consistent with calls for improved computer- and calendar-aided concurrency measurement techniques [8]. Its Web-based implementation allows for real-time logical evaluations, which are not possible with the other methods and they improve data quality and are accessible in a variety of locations by a range of devices. At multiple analysis levels, we assess the agreement of concurrency prevalence measures from this technique with those computed based on the date overlap and direct question methods.

## Methods

### Study Design

Data are from participants’ baseline responses in a 12-month prospective online study of HIV behavioral risks among MSM in the United States, described previously [15,37,38]. Internet-using MSM were recruited from August to December 2010 through selective placement of banner advertisements on social networking websites. Eligibility criteria for participation in the baseline questionnaire were being male, age at least 18 years, and having a male sex partner in the past 12 months. Following online screening and consent, participants completed a 60-minute questionnaire, developed in and hosted on SurveyGizmo 2.6 [39]. The study was reviewed and approved by the Institutional Review Board of Emory University (IRB #00031326).

### Dyadic Data Collection

**Figure 2:**
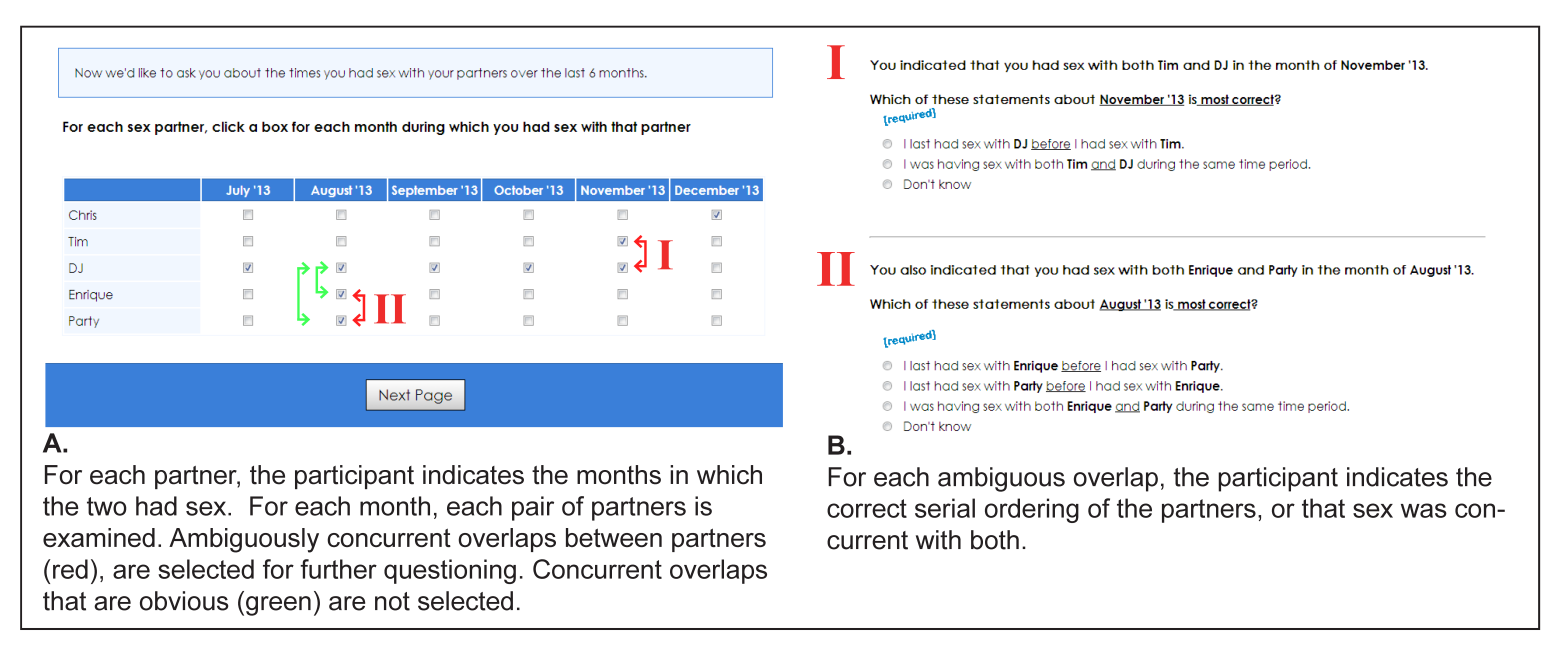
Screenshots of the online partnership timing module illustrating the method for offering follow-up clarification questions.

As part of the online questionnaire, participants who had ≥1 sex partner in the 6 months before the interview were asked to provide nicknames for up to 5 of their most recent anal, oral, or vaginal sex partners within the previous 6 months. This was followed by a novel partnership-timing module, described previously [15,40], which was designed to interactively collect data on concurrency in a method that improved on the existing, passive date overlap and direct question methods. Participants were provided a calendar-like grid of check boxes that displayed the previous 6 months in columns, and partner nicknames on the rows. Participants were asked to indicate the months in which they had sex with each partner (Figure 2). A response pattern that showed 2 or more common months of sex between 2 partners resulted in the triad being later classified as concurrent, consistent with all date overlap techniques. In the case where responses indicated a tie, follow-up direct questions (Figure 2) were asked to clarify whether the participant was with the 2 partners serially or concurrently during the indicated month. This method inherits the easier recall afforded by month resolution dates and direct questioning approaches, but gains the unambiguous sequencing information provided by day resolution dates [40]. Recall is further aided by the ability to visualize all partnerships simultaneously on a calendar, rather than report timing per partnership [8,20]. The partnership-timing module additionally enables concurrency measurement at the individual, dyad, and triad levels. This module has been implemented in multiple studies, with questionnaires administered via desktop computers, laptop computers, and iPads. A demonstration of the partnership-timing module is available [41].

Following the partnership-timing module, participants completed an in-depth demographic and behavioral inventory for each partner. For repeat, rather than 1-time (1-off) sex partners, standard direct concurrency questions were asked [23], along with questions about the partnership’s first and last dates of sex. To help alleviate common problems with missing or invalid dates [19], a flexible series of date questions were asked, with tight logical controls applied. Initially, month/year resolution dates were requested, but participants could opt-in to provide the exact date, if known. If the month was unknown, participants were prompted to select quarters of the year and were shown reminders of familiar events during those seasons to aid recall. If the year of first sex was unknown, ranges of years in the past were provided. Out-of-sequence or invalid (ie, future dates or last sex >6 months prior to interview) first and last sex dates (or approximate dates/quarters) were detected in real time. Participants were then shown their logical error and prompted for correction. Due to the multiple allowances for indicating partial/unknown responses, the date questions could collectively be set as required, further reducing the potential for missing data.

### Focus Group and Facebook Pilot

In December 2009, a focus group with 13 MSM was conducted to evaluate the partnership-timing module in comparison to the direct question and date-based data collection methods. A high degree of acceptability was indicated for the partnership-timing module, which participants felt facilitated recall more than the date collection. Focus group feedback resulted in refinements to the partnership-timing module placement and follow-up question wording. An additional online pilot study was conducted with 1077 Facebook-recruited MSM, with the purpose of testing and refining the questionnaire’s logic.

### Concurrency Measures

Measures of concurrency were calculated at the triadic, dyadic, and participant levels. Each unique combination of a respondent and 2 reported sex partners comprised a triad; each respondent could contribute 0-10 triads based on the number of sex partners in the past 6 months about whom he reported information (5 partners choose 2 triads=10 triads total). Triads were considered concurrent using partnership-timing module responses if the months of sex with both partners overlapped by ≥2 months (criterion A), if they overlapped by 1 month and 1 partner’s interval entirely contained the 1 month relationship of the other partner (criterion B), or based on a response to the clarification questions that affirmed concurrency for a 1-month tie (criterion C). Using the date information for each partner described, triadic date overlap was evaluated by the 3 methods described previously: exact date overlap, month resolution date overlap excluding ties (using criteria A and B) [17,19,21,25], and including ties [20,21,23,25].

At the dyad level, concurrency was classified using direct question responses, dichotomized at ≥1 other sex partners during the relationship being queried [23]. These triadic and dyadic measures were summarized by participant to yield individual-level binary measures of the cumulative occurrence of any concurrency in the previous 6 months.

Lastly, we computed UNAIDS measures of the point prevalence of concurrency before the interview [8,19]. The questionnaire’s 6-month recall period precluded its calculation at 6 months before interview; instead, 5 and 3 months were chosen to represent the closest time to 6 months and the midpoint of the recall period, respectively [25].

### Analytical Methods

We previously described concurrency among 3471 participants who completed the partnership-timing module [15] and in this report we include the 2737 who completed the partner inventory for all partners (2737/3471, 78.85%), consistent with recommendations for concurrency outcome computation [8] For this restricted sample, we computed the distribution of demographic characteristics. Next, the prevalence of individual-level concurrency was computed for all concurrency measures. The percent agreement of concurrency classifications was computed pairwise between the partnership-timing module and the other methods (direct question, all 3 date overlap, and both UNAIDS outcomes). Agreement in excess of chance was assessed by the kappa statistic (κ) and its 95% confidence interval (CI); values from .80-.99 indicate very high, .61-.80 good, .40-.60 moderate, and 0-.40 low agreement [42]. The degree to which discordant concurrency classifications favored the partnership-timing module was quantified by the matched odds ratio (matched OR) and its 95% CI; values >1 indicate more concurrency classified by the partnership-timing module, whereas those <1 indicate more concurrency by the comparison method.

Several participant subsets were examined to further resolve the partnership-timing module’s ability to accurately classify concurrency. To understand whether limiting the partnership inventory to 5 partners constrained concurrency measured by the partnership-timing module relative to the direct method (which has no upper bound), we compared these 2 methods after excluding participants who reported >5 total partners in the previous 6 months. Because participants with only 1 sex partner are automatically classified as not concurrent by all measures, thereby inflating their agreement, we next performed these computations restricted to participants with multiple sex partners [33]. Dates of sex were not collected for 1-time sex partners, which may potentially lower agreement for the date-based methods. To address this, we conducted an analysis restricted to participants who reported multiple and exclusively repeat sex partners, and later discuss the role of 1-time partners in the agreement of concurrency measures.

We additionally assessed the prevalence and agreement of these measures at the dyad and triad levels of analysis. This is because these levels are the ones at which concurrency data are primarily collected (dyadic by direct question, triadic by date methods) and these levels contribute to understanding different aspects of concurrency.

## Results

Among the 2737 participants who completed the partnership inventory, 55.97% (1532/2737) identified as white non-Hispanic, 16.00% (438/2737) as black non-Hispanic, 14.32% (392/2737) as Hispanic, and 13.70% (375/2737) as other race/ethnicity. The median age was 27 years (IQR 22-38) and a median of 2 sex partners (IQR 1-5) was reported in the previous 6 months.

Table 1 displays individual-level prevalence measures of concurrency in the previous 6 months. Using the partnership-timing module, 41.07% (1124/2737) of participants reported at least 1 concurrent triad and thus had concurrent partners. More individual concurrency was identified using the direction question (49.33%, 1333/2702) and lower levels were classified using the date-based measures. The pairwise agreement between the partnership-timing module and the other concurrency measures is displayed in Table 1. Overall, a large degree of agreement was observed (agreement range: 64.82%-85.60%), although substantial variation was seen in agreement that was in excess of chance (κ range .14-.71).

The most agreement was observed with the direct question technique, with 85.60% and a kappa of .71, although the direct question method significantly classified more concurrency (matched OR 0.27, 95% CI 0.21-0.34, *P*<.001). The exclusion of 388 participants with more than 5 total partners reduced both methods’ concurrency prevalences by 7%, but resulted in a negligible change in their agreement (87% agreement, κ=.72).

Concurrency prevalences were 27.52% (753/2736) and 25.73% (704/2736) using overlapping day and month resolution (excluding ties) date measures, respectively. Although these levels were less than that detected with the partnership-timing module, these date measures had nearly identical and moderate agreement with the module (79% agreement, κ=.55). Where the methods differed, the partnership-timing module was more than 4 times more likely to classify individual concurrency (matched OR 4.8 and 6.8 compared to day- and month-level dates methods, respectively). A 32.64% (893/2736) concurrency prevalence was measured by overlapping month-level dates that included ties. Levels of agreement with the partnership-timing module were similar to those of the other date measures, although a lower matched OR of 2.6 (95% CI 2.1-3.1) was observed.

The lowest levels of concurrency were observed using the 2 modified UNAIDS point prevalence measures. A total of 5.34% (146/2736) of participants reported concurrent partnerships at 5 months before interview, and 6.76% (185/2736) did so at 3 months beforehand. Similarly, the agreements between these measures and the partnership-timing module were lowest (κ=.14 and .17 at 5 and 3 months, respectively). Additionally, the 2 modified UNAIDS measures had high agreement with one another (96.97% agreement, κ=.73). To assess the degree to which the use of month-level dates with the exclusion of ties might have diminished the UNAIDS measure estimates, we calculated these point prevalences using day resolution date information and found prevalences of 16.11% (411/2737) and 17.32% (474/2737) at 5 and 3 months, respectively.

**Table 1:** Individual-level concurrency by partnership-timing module and alternative measures of concurrency among 2737 men who have sex with men.

Concurrency measure	Concurrency prevalence		Agreement with partnership-timing module			
	Concurrent, n (%)	Missing, n	Agreement, n (%)	Both methods concur, n	κ (95% CI)	Matched OR (95% CI)
Partnership-timing module	1124 (41.07)	0	—	—	—	—
Direct question	1333 (49.33)	35	2313 (85.60)	1026	.71 (.69-.74)	0.27 (0.21-0.34)
Date overlap, day resolution^a^	753 (27.52)	1	2171 (79.35)	656	.55 (.52-.58)	4.8 (3.9-6.0)
Date overlap, month resolution, excluding ties	704 (25.73)	1	2172 (79.39)	632	.55 (.52-.58)	6.8 (5.4-8.8)
Date overlap, month resolution, including ties	893 (32.64)	1	2209 (80.74)	745	.59 (.56-.62)	2.6 (2.1-3.1)
UNAIDS, 5 months before interview^b^	146 (5.34)	1	1746 (64.82)	140	.14 (.2-.16)	164.0 (78.6-405.5)
UNAIDS, 3 months before interview	185 (6.76)	1	1781 (65.10)	177	.17 (15-.20)	118.4 (62.1-255.6)

^a^Date overlaps measures exclude 1-time partners, for whom dates of sex were not asked.

^b^UNAIDS point prevalence measures modified to 5 and 3 months from typical 6 months.

Table 2 displays these same metrics for those participants who reported ≥1 sex partner. Among these participants, the prevalence of concurrency as measured by the partnership-timing module was 60.30% (1124/1864). As anticipated, this restriction also caused all other prevalence measures to increase (range: direct question 70.09%-UNAIDS 5 months 7.84%) and their agreement with the partnership-timing module to decrease (κ range: direct question .59-UNAIDS 5 months .09). For this subgroup, the agreement between the direct question and the tie-inclusive overlapping dates methods were fair (κ=.44), similar to that reported among US heterosexuals (κ=.40) [20] and higher than that among Malawian heterosexuals (κ≈.23) [21].

A further restriction to participants with exclusively repeat partners is shown in Table 2. A 73.94% (227/307) concurrency prevalence was observed using the partnership-timing module. High and similar levels of agreement were observed for the direct question and date overlap methods compared to the partnership-timing module (agreement range 86.60%-90.13%, κ range .65-.72). Despite high agreement, very low matched OR were seen for the direct question (matched OR 0.07, 95% CI 0.01-0.26) and tie-inclusive date overlap methods (matched OR 0.03, 95% CI 0.00-0.15). In contrast, poor agreement was seen between the UNAIDS and partnership-timing module measures (% agreement: 48.69%, 149/306 and 50.98%, 156/306, κ range .17 and .20 for 5 and 3 months, respectively).

The measurement of concurrency at each method’s primary unit of measurement is shown in Table 3. Participants indicated concurrent partners during 56.71% (2667/4703) of partnerships involving repeat partners using the partnership-timing module. Using the direct question module, this was 67.29% (2927/4703) with a substantial level of agreement (83.66%, κ=.66). Discordantly classified partners were 5 times as likely to be considered concurrent by the direct question method (matched OR 0.20, 95% CI 0.16-0.24). Among triads involving 2 repeat partners, 63.65% (1879/2962) of those involving 2 repeat partners were concurrent. Agreement was consistent and moderate with the 3 overlapping dates measures (agreement range 78.62%-80.96%, κ range .48-.59). By the tie-inclusive overlapping dates method, triadic concurrency prevalence was high (81.77%, 2378/2962), with high tendency to classify discrepant triads as concurrent compared to the partnership-timing module (matched OR 0.08, 95% CI 0.06-0.10).

**Table 2:** Individual-level concurrency by partnership-timing module and alternative measures of concurrency among subsets of men who have sex with men.

Concurrency measure		Concurrency prevalence		Agreement with partnership-timing module			
		Concurrent, n (%)	Missing, n	Agreement, n (%)	Both methods concur, n	κ (95% CI)	Matched OR (95% CI)
**Participants with multiple partners (n=1864)**							
	Partnership-timing module	1124 (60.30)	0	—	—	—	—
	Direct question^a^	1291 (70.09)	22	1495 (81.16)	1026	.59 (.55-.63)	0.31 (0.24-0.40)
	Date overlap, day resolution^b^	753 (40.42)	1	1298 (69.67)	656	.42 (.38-.45)	4.8 (3.9-6.0)
	Date overlap, month resolution, excluding ties	704 (37.79)	1	1299 (69.73)	632	.42 (.39-.46)	6.8 (5.4-8.8)
	Date overlap, month resolution, including ties	893 (47.93)	1	1326 (71.18)	745	.44 (.40-.48)	2.6 (2.1-3.1)
	UNAIDS, 5 months before interview^c^	146 (7.84)	1	873 (46.86)	140	.09 (.08-.11)	164.0 (78.6-405.5)
	UNAIDS, 3 months before interview	185 (9.93)	1	908 (48.74)	177	.12 (.10-.14)	118.4 (62.1-255.6)
**Participants with multiple and exclusively repeat partners (n=307)**							
	Partnership-timing module	227 (73.94)	0	—	—	—	—
	Direct question^a^	250 (82.24)	3	274 (90.13)	222	.72 (.62-.81)	0.07 (0.01-0.26)
	Date overlap, day resolution^b^	231 (75.49)	1	268 (87.58)	210	.67 (.57-.77)	0.81 (0.42-1.5)
	Date overlap, month resolution, excluding ties	224 (73.20)	1	265 (86.60)	205	.65 (.56-.75)	1.2 (0.62-2.2)
	Date overlap, month resolution, including ties	260 (84.97)	1	271 (88.56)	226	.65 (.55-.76)	0.03 (0.00-0.15)
	UNAIDS, 5 months before interview^c^	76 (24.84)	1	149 (48.69)	73	.17 (.12-.23)	51.3 (18.6-203.8)
	UNAIDS, 3 months before interview	83 (27.12)	1	156 (50.98)	80	.20 (.14-.26)	49.0 (17.7-194.6)

^a^3% (n=42) of those who indicated concurrency by the direct question also named only 1 sex partner. Accordingly, the 42 individuals are included in Table 1, but are excluded from the subsets of participants with multiple partners in Table 2.

^b^Date overlaps measures exclude 1-time partners, for whom dates of sex were not asked.

^c^UNAIDS point prevalence measures modified to 5 and 3 months from typical 6 months.

**Table 3:** Dyad- and triad-level concurrency by partnership-timing module and alternative measures of concurrency among subsets of men who have sex with men.

Concurrency measure		Concurrency prevalence		Agreement with partnership-timing module			
		Concurrent, n (%)	Missing, n	Agreement, n (%)	Both methods concurrent, n	κ (95% CI)	Matched OR (95% CI)
**Dyad level, repeat partners (n=4703)**							
	Partnership-timing module	2667 (56.71)	0	—	—	—	—
	Direct question	2927 (67.29)	353	3639 (83.66)	2334	.66 (.64-.68)	0.20 (0.16-0.24)
**Triad level, repeat partners (n=2962)**							
	Partnership-timing module	1879 (63.65)	10	—	—	—	—
	Date overlap, day resolution	1986 (68.29)	54	2291 (78.76)	1608	.53 (.50-.56)	0.63 (0.54-0.74)
	Date overlap, month resolution, excluding ties	1842 (63.34)	54	2355 (80.96)	1568	.59 (.56-.62)	1.0 (0.86-1.2)
	Date overlap, month resolution, including ties	2378 (81.77)	54	2287 (78.62)	1802	.48 (.45-.52)	0.08 (0.06-0.10)

For the set of participants with multiple partners considered in Table 2, we examined individual-level correlates of agreement with the partnership-timing module (Table 4). Agreement in classifying concurrency between each of the 6 alternative methods and the partnership-timing module did not significantly vary by race/ethnicity, age, or annual income (kappa values not significantly different). Agreement significantly differed by education level for the day resolution and month resolution, excluding ties methods, with those reporting some college education having the highest agreement.

**Table 4:** Individual-level concurrency and correlates of agreement between the partnership-timing module and alternative measures of concurrency among men who have sex with men reporting multiple partners.

Concurrency measure	Participants, n (%) (n=1864)		Concurrency prevalence, %	Agreement with partnership-timing module, κ					
				Direct question	Date overlap			UNAIDS	
					Day resolution	Month resolution, excluding ties	Month resolution, including ties	5 months before interview	3 months before interview
**Race/ethnicity**										
	White	1035 (55.53)	62.03	.60	.41	.41	.44	.08	0.11
	Black	309 (16.58)	58.58	.52	.42	.45	.46	.12	0.12
	Hispanic	283 (15.18)	57.60	.55	.42	.42	.41	.10	0.13
	Other	237 (12.71)	58.23	.66	.43	.44	.43	.12	0.14
	*P* value			*P*=.16	*P*=.98	*P*=.85	*P*=.93	*P*=.57	*P*=.84
**Age (years)**									
	18-19	195 (10.46)	47.69	.61	.47	.48	.46	.11	0.11
	20-24	529 (28.38)	55.77	.60	.43	.43	.46	.07	0.12
	25-29	344 (18.45)	61.34	.55	.37	.37	.37	.07	0.09
	30-39	376 (20.17)	60.90	.62	.39	.39	.47	.08	0.11
	40-49	273 (14.65)	72.53	.54	.38	.41	.40	.12	0.13
	≥50	147 (7.89)	66.67	.52	.46	.46	.42	.12	0.13
	*P* value			*P*=.67	*P*=.67	*P*=.64	*P*=.64	*P*=.42	*P*=.83
**Education**									
	College/postgraduate	748 (40.17)	65.51	.59	.36	.35	.38	.08	0.09
	Some college/associate degree	761 (40.87)	56.24	.60	.49	.50	.49	.13	0.15
	High school or GED	298 (16.00)	54.55	.52	.37	.42	.44	.07	0.11
	Less than high school	55 (2.95)	59.06	.70	.42	.46	.41	.09	0.15
	*P* value			*P*=.37	*P*=.02	*P*=.004	*P*=.11	*P*=.06	*P*=.05
**Annual income**									
	≤$14,999	574 (32.50)	54.53	.55	.41	.43	.43	.09	0.11
	$15,000-$39,999	543 (30.75)	59.48	.61	.41	.43	.42	.09	0.13
	$40,000-$74,999	383 (21.69)	65.80	.62	.41	.40	.43	.09	0.11
	≥$75,000	266 (15.06)	71.80	.53	.40	.36	.44	.10	0.12
	*P* value			*P*=.41	*P*=.99	*P*=.62	*P*=.99	*P*=.95	*P*=.83

## Discussion

In this comparison of extant concurrency measures and measures derived from a new partnership-timing module, a wide range was seen in the overall prevalence of concurrency among our sample of MSM, which may help to explain the sizeable variability seen in published estimates of concurrency prevalence among MSM [7,16]. Overall, the observed levels of agreement between the partnership-timing module with date overlap and direct question cumulative prevalence measures are higher than we and others have found among these latter 2 types of measures [20,21]. Further, the prevalences of concurrency measured by the partnership-timing module were between those resulting from these 2 measurement types. This is consistent with our expectations, as aspects of the partnership-timing module are borrowed from these techniques.

The greatest degree of agreement was seen with the direct question measures, which consistently yielded the highest frequency of concurrency consistent with what others have reported [21]. That this highest prevalence was seen despite restricting to individuals with less than 5 partners corresponds to either better concurrency detection abilities of the direct question method or its inadequate validity. Because direct question concurrency was seen among 3%-7% of those with 1 named partner, this approach likely has limited specificity. Others have attributed this to underreporting in partner histories and priming effects of the direct questions [21,26,43]; however, we observed this phenomenon even more frequently when considering 6-month partner counts (not dyadic section partners) provided earlier in the questionnaire. Due to potential overclassification and the previously described analytical limitations for the direct question measures, the high levels of agreement between the techniques and that the partnership-timing module retains direct questioning where critical, we feel the partnership-timing module seems like an appropriate alternative to the direct question approach.

More individuals were classified as having concurrent partners using the partnership-timing module than with all date overlap methods. Examining those with exclusively repeat partners, agreement was markedly improved. Some of this is likely explained by our study’s limitation of not asking dates of 1-time partners, who represented almost half of partners described in this sample (45%, 3907/8610), and may be involved in a substantial proportion of concurrent triads among MSM. This pattern is less common and has been generally disregarded as unimportant for concurrency-related HIV transmission in other contexts [8]. However, the role of 1-time partnerships in MSM concurrency transmission is yet to be determined and may be broader, given the greater HIV transmission risks per sexual act and the documentation of transmission bursts among MSM [4,44]. The inclusion of sex date for 1-time partners would increase date overlap measure prevalence, yet it is unclear whether the agreement of these measures would be substantially improved compared to the partnership-timing module for several reasons. Poor recall for ongoing partnerships has led to the seldom use of day resolution concurrency measures. Although the enhanced date collection methods used may have improved date recall and quality, data quality would likely be worse for 1-time partners. The more commonly used month resolution measures showed a greater disparity in the degree of concurrency detected, owing to differential classification of repeat “tie” partnerships of short duration but within 1 calendar month. The influx of 1-time partners would necessarily inflate the number of 1-month partnerships and cause the agreement of the 2 month resolution measures to diverge further, representing upper and lower bounds of the true date-based concurrency estimate. Indeed, the partnership-timing module was designed precisely to alleviate this ambiguity among MSM partnerships.

Relatively low levels of concurrency were detected by the UNAIDS-style point prevalence measures at 5 and 3 months, suggesting low sensitivity in this population. The 2 prevalence measures were consistently similar, implying that the precise time-point may be arbitrary, and suggesting a plausible range for the 6-month indicator, if it were computable. A portion of the low detection may be explained by the exclusion of 1-time partners. However, the UNAIDS method always excludes ties and many 1-time partnerships would manifest as single-month ties, rather than being fully “contained” within another multimonth partnership. The degree to which classification was impeded by excluding ties was quantified by substituting day resolution point prevalence (a nonstandard measure), which resulted in modestly increased classification and a nearly identical change in estimate to that observed among Kenyan heterosexuals [25]. This method by definition excludes all 1-time partners, except for those on the exact day being assessed, and the inclusion of 1-time partner dates would not change the estimates of 16% and 17%. Nonetheless, the month resolution measures we found are in the range of those reported among most samples of sub-Saharan African heterosexual men [21,33,45]. This implies a false equality in concurrency patterns between these 2 populations, given the documentation of substantially different concurrency cumulative prevalence [7,15], which is likely partially due to differences in partner duration among MSM. The UNAIDS measure accordingly appears to be ill suited for detecting concurrency among MSM in either surveillance or research contexts, unless momentary degree is specifically required for dynamic modeling.

In addition to the measure-specific limitations discussed, this report is subject to several broad limitations. Participants were sampled from social network sites and may not represent the broader MSM population in the United States, although recent analyses have suggested the relative comparability of men sampled through social websites compared to through MSM venues [46]. Participant dropout in the partnership inventory, likely owing to the nonincentivized and online nature of the study, may have biased observed results, specifically lowering concurrency estimates because those with more partners were more likely to not complete the questionnaire. We earlier reported 45% individual-level concurrency among 3519 men who began, but did not necessarily complete, this section [15]. This is similar to the 41% observed in this report and partly allays these concerns. We also recognize that concurrency measured on subsets, such as those with multiple partners, do not necessarily make valid population-wide estimates because their validity is tied to the occurrence of those subsets. These subsets should be used only to weigh the relative merits of measurement approaches. Last, we have only considered the performance of these concurrency tools and measures among MSM. In other at-risk populations, particularly those with longer-term concurrently overlapping relationships, fewer differences between measures are expected. Nonetheless, the desire to conduct analysis at other levels should be considered in selecting the appropriate concurrency measure. Compared to other measurement approaches, the partnership-timing module requires that more complex computer programming logic be executed in real time to be implemented. This may impede its application in some surveillance contexts. Yet as technologically enhanced data collection modalities become sophisticated and normative, this limitation will become less prominent.

Across a range of comparisons, the partnership-timing module showed strong concurrency detection ability and agreement with extant measures among an online sample of MSM. The technique overcomes known limitations of other concurrency collection approaches and measures, and may be well suited to MSM partnership patterns. Furthermore, its placement before detailed partnership questions may help to avoid priming participants for socially desired responses [26], while providing the benefit of generally reorienting participants to their sexual histories. Further research of concurrency among MSM should consider the incorporation of this new measurement technique.

## Abbreviations

HIV: human immunodeficiency virus
MSM: men who have sex with men
UNAIDS: The Joint United Nations Programme on HIV/AIDS
